# Deutscher Rheumatologiekongress virtuell – erfolgreich tagen trotz Pandemie

**DOI:** 10.1007/s00393-021-00997-2

**Published:** 2021-04-20

**Authors:** Jutta G. Richter, Gamal Chehab, Johannes Knitza, Anna Krotova, Matthias Schneider, Anna Julia Voormann, Hendrik Schulze-Koops, Christof Specker

**Affiliations:** 1 Poliklinik, Funktionsbereich und Hiller Forschungszentrum für Rheumatologie, Universitätsklinikum Düsseldorf, Medizinische Fakultät, Heinrich-Heine-Universität Düsseldorf, Moorenstr. 5, 40225 Düsseldorf, Deutschland; 2 Medizinische Klinik 3 – Rheumatologie und Immunologie, Universitätsklinikum Erlangen, Friedrich-Alexander-Universität Erlangen-Nürnberg (FAU), Erlangen, Deutschland; 3 Rheumatologische Fortbildungsakademie, Deutsche Gesellschaft für Rheumatologie e. V., Berlin, Deutschland; 4 Sektion Rheumatologie und Klinische Immunologie, Medizinische Klinik und Poliklinik IV, Klinikum, Universität München, München, Deutschland; 5 Klinik für Rheumatologie & Klinische Immunologie, Evangelisches Krankenhaus, Kliniken Essen-Mitte, Essen, Deutschland

**Keywords:** Digitale Rheumatologie, COVID-19-Pandemie, Virtuelle Veranstaltung, Akzeptanz, Digitale Fortbildung, COVID-19 pandemic, Virtual meeting, Digital rheumatology, Acceptance, Digital Continuing Medical Education

## Abstract

**Hintergrund:**

Die COVID-19-Pandemie führte im Jahr 2020 dazu, dass der Jahreskongress der Deutschen Gesellschaft für Rheumatologie (DGRh) als „Deutscher Rheumatologiekongress virtuell“ durchgeführt wurde.

**Fragestellung:**

Wie wird der „Deutsche Rheumatologiekongress virtuell“ angenommen und welche Optimierungsmöglichkeiten bieten sich für die Zukunft?

**Material und Methode:**

Die registrierten Teilnehmer wurden gebeten, an einer Online-Kongressevaluation teilzunehmen.

**Ergebnisse:**

Von 2566 Kongressteilnehmern nahmen 721 an der Evaluation teil. Die Mehrheit (80,2 %) war mit der Veranstaltung insgesamt zufrieden oder sehr zufrieden. Das Format wurde als für den kollegialen Austausch weniger geeignet angesehen. Die verwendete Technik wurde überwiegend als leicht bedienbar und problemlos zugänglich beschrieben. Die ausgewählten Themen des Kongresses entsprachen den Erwartungen von 89 % der Teilnehmer. Die präsentierten Inhalte wurden von 85,2 % der Teilnehmer als für ihre Tätigkeit relevant eingestuft. Eine deutliche Mehrheit der Teilnehmer (85,3 %) würde es begrüßen, die Kongressinhalte dauerhaft und flexibel „on demand“ abrufen zu können.

**Diskussion:**

Insgesamt konnte ein gut akzeptiertes Format für die Durchführung des „Deutschen Rheumatologiekongresses virtuell“ gefunden werden. Optimierungsaspekte konnten aufgezeigt werden, diese können bei der Umsetzung weiterer (digitaler) Kongresse Berücksichtigung finden. Die Ergebnisse dieser Arbeit geben Aufschluss darüber, wie die DGRh virtuelle und/oder hybride Konferenzen in der Zukunft gestalten kann, um den Interessen und Wünschen der Teilnehmer zu entsprechen.

## Abstract (en)

**Background:**

In 2020 the COVID-19 pandemic led to the annual meeting of the German Society for Rheumatology (DGRh) being conducted as the virtual German Rheumatology Congress.

**Aim:**

How is the virtual German Rheumatology Congress accepted and what are the possibilities of optimization for the future?

**Material and method:**

The registered participants were asked to take part in an online congress evaluation.

**Results:**

Of 2566 congress attendees, 721 participated in the evaluation. The majority (80.2%) were (very) satisfied with the event overall. Compared to the traditional format collegial exchange was perceived as worse using the virtual approach. The technology platform was predominantly described as easy to use and easily accessible. The selected topics of the congress met the expectations of 89% of the participants. The presented contents were estimated to be relevant for their professional activities by 85.2% of the participants. The majority of participants (85.3%) would welcome the congress contents to be permanently available on demand.

**Discussion:**

Overall, the participants appreciated the virtual format of the German Rheumatology Congress. Optimization aspects could be shown and these could be considered in the implementation of further (digital) congresses. The results of this work provide suggestions for improvement on how the DGRh can meet and exceed the needs of participants in organizing a virtual or hybrid conference in the future.

## Hintergrund

Durch die COVID-19-Pandemie waren im Rahmen des Infektionsschutzgesetzes sowie der verordneten Kontakt- und Mobilitätsbeschränkungen seit März 2020 Vor-Ort-Fortbildungsveranstaltungen mit größerer Personenanzahl nicht mehr möglich. Geplante Workshops, Seminare, aber auch Kongresse, wurden konsekutiv auf digitale Formate umgestellt. Inzwischen wurden auch „Guidelines“ publiziert, die erläutern, wie virtuelle Kongresse bestmöglich geplant werden können [1]. Zur Umsetzung der digitalen Fortbildungsformate werden unter anderem sog. „Softwarelösungen für Videokonferenzen“ genutzt, wie sie beispielsweise von Zoom (San José, Ca, USA; https://zoom.us/de-de/meetings.html) und Cisco Webex (Milpitas, Ca, USA; https://www.webex.com/de/video-conferencing.html) professionell angeboten werden. Das daraus resultierende Online-Format wurde als „die neue Form der Fortbildung“ schnell angenommen, digitale Meetings gelten inzwischen als akzeptiert und etabliert [2].

Da auch medizinische Fachgesellschaften in der Verantwortung sind, eine Verbreitung der SARS-CoV-2-Infektion durch große Menschenansammlungen zu verhindern, wurde als einer der ersten rheumatologischen Kongresse die Jahrestagung der European League Against Rheumatism (EULAR) im Juni 2020 in ein virtuelles Format überführt [3].

Aus Sicht der Deutschen Gesellschaft für Rheumatologie e. V. (DGRh) leitete sich aus der fortbestehenden Pandemie die Notwendigkeit ab, den für September 2020 traditionell als Vor-Ort-Kongress geplanten Jahreskongress ebenfalls auf ein überwiegend virtuelles Format umzustellen. Daran wurde ab Juni 2020 vonseiten der DGRh und der Rheumatologischen Fortbildungsakademie („Rheumaakademie“) intensiv gearbeitet und an alle Beteiligten kommuniziert. Basierend auf der Programmstruktur wurden verschiedenen Formate für die Darstellung der Themen genutzt: Vorträge wurden vielfach voraufgezeichnet, teils um eine Live-Diskussion ergänzt. Andere Sitzungen waren ausschließlich live zu verfolgen. Insbesondere aktuell zu diskutierende und vielschichtige Themen wurden in den stärker interaktiven Formaten angeboten.

In Zusammenarbeit mit der Rheumaakademie entwickelten einige regionale Rheumazentren hybride Konzepte, um den persönlichen wissenschaftlichen Austausch und Diskussionen auf regionaler Ebene zu ermöglichen und diese in den virtuellen Kongress hineinzutragen. Sechs DGRh-Rheumazentren (Dresden/Chemnitz, Heidelberg, München e. V., Rhein-Main e. V., Rhein-Ruhr e. V., Leipzig e. V.) organisierten dazu ein sog. „Public Viewing“ vor Ort. Um regulatorische Vorgaben (z. B. Hygieneregeln) zu erfüllen, wurde ein teils erheblicher Aufwand betrieben, welcher nur mit finanzieller Unterstützung der pharmazeutischen Industrie umgesetzt werden konnte.

Die Evaluation der Jahreskongresse der DGRh hat bereits eine lange Tradition. Sie wurde zuletzt durch die Rheumaakademie kontinuierlich durchgeführt und zur ständigen Verbesserung und Weiterentwicklung der Kongressinhalte genutzt. Teilnehmerzahlen und soziodemografische Profile wurden beispielsweise auch schon für wissenschaftliche Zwecke genutzt [4].

Aus dem neuen digitalen Kongressformat leitete sich aus Sicht des Vorstandes und der Kommission Digitale Rheumatologie der DGRh die Notwendigkeit ab, dieses besonders zu evaluieren. Der Vorstand beauftragte die Kommission Digitale Rheumatologie der DGRh, ein Evaluationskonzept zu entwickeln und dieses für eine Onlinebefragung umzusetzen. Dieser Aufgabe gingen die Autoren kurzfristig nach. Vorrangiges Ziel war zu analysieren, ob und wie das neue technische Format den Aufgaben eines Kongresses gerecht wird und Akzeptanz bei den Teilnehmern findet. Optimierungsmöglichkeiten für die Zukunft sollten identifiziert werden.

## Material und Methode

Üblicherweise werden die Kongressthemen und die sog. Convenor der einzelnen Sessions für den Jahreskongress der Rheumatologie etwa 1 Jahr vor der Veranstaltung durch das wissenschaftliche Programmkomitee festgelegt. Die Convenor gestalten bis zum Frühjahr des Veranstaltungsjahres die Sessions aus (Themen und Dauer der einzelnen Vorträge, Referenten). Die Entscheidung, den Kongress rein virtuell durchzuführen, fiel im Juni 2020.

Nach dieser Entscheidung wurde der Deutsche Rheumatologiekongress als „Rheumatologiekongress virtuell“ in Kooperation mit der Fima M‑Events Cross Media GmbH als reiner online Kongress gestaltet. Die Entscheidung für die Firma wurde aus praktischen und ökonomischen Gründen getroffen. Vorteilhaft erwies sich dabei, dass zwischen der M‑Events und der Rheumaakademie bereits eine gute Zusammenarbeit bestand und die Firma einer der spezialisierten Anbieter auf dem Gebiet professioneller technischer Begleitung von Kongressen medizinischer Fachgesellschaften ist. Folgende Session-Formate wurden als geeignet eingestuft: „Live“, „On Demand“, „Fishbowl“, „ePosterwalk“ und „interaktiv“. Ziel war es, anstelle einer reinen Vortragsveranstaltung abwechslungsreiche Formate anbieten zu können und den Teilnehmenden möglichst viel Gelegenheit zur Interaktion zu geben. Dafür wurden – nach Verschlankung des Programms und auf Basis inhaltlicher Erwägungen durch das Programmkomitee – Vorträge aufgezeichnet, live gehalten – mit der Möglichkeit für Fragen im Chat, im Chat kommentiert, live mit den Vorsitzenden diskutiert und auch – im sog. „Fishbowl-Format“ – live von einer Gruppe diskutiert und kommentiert. Für die ePosterwalks war neben den digitalen Postern eine Vorabvideoaufzeichnung als Kurzpräsentationen der Poster gewünscht, diese standen während des gesamten Kongresses zur Verfügung.

Die Teilnehmer registrierten sich für den Kongress ausschließlich online. Mit den elektronisch zugesandten Login-Daten konnte diese sich über eine Anmeldeoberfläche in den virtuellen „Live Kongress“ einwählen und so an den angebotenen Sessions teilnehmen sowie im Live-Chat mitdiskutieren.

Die verschiedenen Sessions standen angemeldeten Teilnehmern über den „Live Kongress“ hinaus bis zum 31.12.2020 auf Abruf online zur Verfügung, sofern die Vortragenden und Autoren ihre ausdrückliche Zustimmung hierzu erteilt hatten und auch keine Rechtsansprüche Dritter dem entgegenstanden.

In ihrem persönlichen Profil konnten die Teilnehmer nach Abschluss des Kongresses ihre persönliche Teilnahmebescheinigung und ihre dokumentierten CME-Punkte herunterladen. In diesem Kontext wurden die Teilnehmer gebeten, an der Kongressevaluation teilzunehmen. Dazu mussten sie auf einen Link klicken, der sie zu der mit dem Tool „QuestionPro“ gestalteten Umfrage führte. Der dort hinterlegte Fragebogen war von den Autoren der vorliegenden Veröffentlichung gemeinsam konzipiert und von den Mitarbeitern der Rheumaakademie umgesetzt worden. Auf eine detailliertere Evaluation auf Ebene einzelner Sessions wurde nach intensiver Diskussion verzichtet, um die Teilnehmer nicht durch zu viele Fragen zu belasten. Die letzte Version wurde durch den Vorstand der DGRh und die Mitglieder der Kommission Digitale Rheumatologie konsentiert.

Zur Einordnung der Antworten wurden neben den Kongress-bezogenen Fragen einige soziodemografische Angaben erbeten. Darüber hinaus gab es neben geschlossenen Fragen auch die Möglichkeit zu Freitextangaben. Diese wurden manuell von den Mitarbeitern der Rheumaakademie kategorisiert. Aufgrund der technischen Möglichkeiten des Umfragetools wurden zudem 5er-Likert-Skalen (1 bis 5) angeboten. Die Daten wurden mit der Statistik- und Analyse-Software IBM SPSS® Statistics Versionen 25 für Microsoft Windows analysiert. Die „Tag-Cloud“ wurde mittels WordArt online auf https://wordart.com erstellt.

Nach persönlicher Rücksprache (JK) mit der Ethikkommission an der Friedrich-Alexander-Universität Erlangen-Nürnberg war für die Befragung kein Ethikvotum notwendig. Die Kongressteilnehmer wurden bei der Kongressregistrierung auf die Verarbeitung personenbezogener Daten hingewiesen, und bei der Kongressevaluation wurde ausdrücklich die Freiwilligkeit einer Teilnahme daran betont.

Die Umfrage war ab dem 09.09.2020 bis zum 31.12.2020 im Kongressportal online gestellt. An der Umfrage konnte theoretisch mehrmals teilgenommen werden. Eine Möglichkeit, doppelte Beantwortungen aus den erhobenen und ausgewerteten Daten zu identifizieren, bestand für die Autoren u. a. aus Datenschutzgründen nicht. Für die vorgestellten Auswertungen wurden Evaluationen genutzt, die im Zeitraum zwischen dem 09.09.2020 und 22.11.2020 eingingen.

## Ergebnisse

Insgesamt wurden im Rahmen des virtuellen Kongresses 75 Sessions angeboten, dabei standen die Formate „Live“ (*n* = 68), „On Demand“ (*n* = 7) zur Verfügung. Das Format „Live“ beinhaltete die Formate „Fishbowl“ (*n* = 3), „ePosterwalk“ (*n* = 13) und „interaktiv“ (*n* = 15).

In diesem Jahr verzeichnete der Kongress 305 angenommene Abstracts, die viele Bereiche der klinischen und Grundlagenwissenschaft abdeckten; 190 wurden direkt als Vorträge im Rahmen des ePosterwalks vorgestellt, sodass dieses Angebot nahezu durchgehend während des gesamten Kongresses mit Live-Inhalten verfügbar war. Während des Kongresses wurden 7 Facebook-Veröffentlichungen unter dem Account https://www.facebook.com/dgrh1927 verzeichnet. Der Account der AG junge Rheumatologen (#rheumadocs) zeigte 37 (re-)tweets Posts bei Twitter und 14 Einträge bei Facebook (https://de-de.facebook.com/rheumadocs/?ref=page_internal).

An dem Kongress nahmen insgesamt 2566 Personen teil, davon waren 630 Mitglieder der DGRh. Zudem nahmen 86 MFA/RFA und 867 Mitarbeiter der pharmazeutischen Industrie teil. Da es keinen Patiententag für Rheumapatienten gab, den sonst der jeweilige Landesverband der Deutschen Rheumaliga anlässlich der Jahrestagung organisiert, waren unter den Teilnehmern des virtuellen Kongresses keine Patienten. Die Public Viewing Aktivitäten der 6 Rheumazentren wurden vor Ort am 10.09.2020 von insgesamt 87 und am 11.09.2020 von insgesamt 128 Personen wahrgenommen.

An der Kongressevaluation nahmen 721 Personen teil (84,4 % DGRh-Mitglieder, 54,8 % weiblich, 0,4 % divers). Die meisten stammten aus Deutschland (86,3 %), es gingen aber auch Antworten aus z. B. Österreich, der Schweiz, Kanada, Japan und den USA ein. Für die Evaluation wurden im Mittel 5,9 ± 21,3 (Median 3,1) Minuten benötigt.

Folgende Berufsgruppen nahmen an der Evaluation teil: Ärzte (74,9 %), medizinische bzw. rheumatologische Fachangestellte (5,1 %), Naturwissenschaftler (3,8 %), Studierende (2,5 %), Vertreter der pharmazeutischen Industrie (11,0 %) und andere (2,8 %). Die selbstberichteten Tätigkeitsfelder zeigten sich wie folgt: Praxis (43,2 %), Universitätsklinik (17,8 %), sonstige Klinik (21,0 %), Labor (0,6 %) und anderes (17,4 %).

Die meisten Evaluierenden waren zwischen 50 und 59 Jahre alt (39,5 %), die übrigen Altersgruppen wurden wie folgt berichtet: < 30 Jahre 4,1 %, 30 bis 39 Jahre 14,4 %, 40 bis 49 Jahre 22,1 %, 60 bis 69 Jahre 17,1 % und > 70 Jahre 2,8 %.

Für die Teilnahme am virtuellen DGRh-Kongress benutzten nach der Evaluation 82,2 % einen Laptop oder Desktop-PC, 12,4 % ein Tablet und 4,2 % ein Mobiltelefon. Dabei zeigte sich, dass Ärzte, RFA/MFA und Studierende überwiegend einen Laptop/Desktop-PC nutzten, während bei Vertretern der pharmazeutischen Industrie vornehmlich ein Tablet zum Einsatz kam (30,3 % dieser Gruppe). Die Kongressplattform war für 78,4 % „problemlos“ oder „eher problemlos“ zugänglich. Die von Ärzten berichtete Zugänglichkeit war nicht vom Tätigkeitsort (Klinik/Praxis) abhängig. Die Software wurde von 80,2 % der Teilnehmer als leicht oder eher leicht bedienbar eingestuft (5er-Likert-Skala 1 [trifft zu] bis 5 [trifft nicht zu]), im Mittel lag die Bewertung bei 1,8 ± 1,0, signifikante Unterschiede zeigten sich bezüglich Alter, Berufsgruppe und Tätigkeitsort nicht.

Der Nutzen interaktiver audiovisueller Elemente (z. B. Quizfragen, interaktive Grafiken) für das Verständnis und den persönlichen Lernzuwachs wurde im Mittel mit 2,3 ± 1,0 eingeschätzt (5er-Likert-Skala sehr hoch [1] – sehr niedrig [5]). Relevante Gruppenunterschiede zeigten sich nicht.

**Figure 1:**
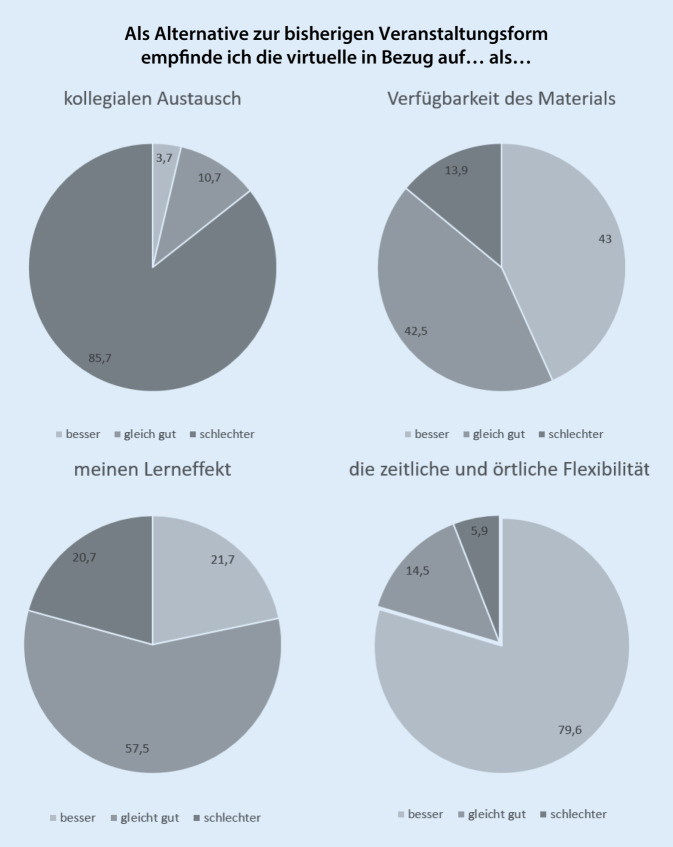

Die Teilnehmer wurden gebeten, das virtuelle Kongressformat mit der bisherigen Veranstaltungsform zu vergleichen. Die Antworten der Gesamtgruppe in Bezug auf 4 hierzu gestellte Fragen zeigt Abb. 1. Sehr häufig wurde das Format für den kollegialen Austausch als schlechter eingestuft: 90,8 % der Vertreter der pharmazeutischen Industrie, 88,9 % der Naturwissenschaftler und 85,8 % der Ärzte und dies unabhängig vom Tätigkeitsort (Uni‑)Kliniken und Praxen (85,5 % respektive 87,9 % und 84,1 %).

Die Verfügbarkeit der Inhalte wurde beim virtuellen Kongress gegenüber dem bisherigen Veranstaltungsformat von Klinikern als „besser“ (48,4 %) bzw. „gleich gut“ (40,7 %) eingeschätzt, 41,9 % bzw. 44,2 % der in Praxen Tätigen teilten dies. Von den RFAs/MFAs stimmten dem mit 25,0 und 52,8 % weniger zu. Diese empfanden mit 22,2 % das inhaltliche Format des virtuellen Kongresses als schlechter gegenüber konventionellen Veranstaltungen, was auf ärztlicher Seite für 10,9 % der in Klinik und 13,9 % der in Praxis Tätigen galt.

Ein Drittel der Studierenden und 29,6 % der Naturwissenschaftler bewerteten ihren Lerneffekt in der digitalen Veranstaltung als schlechter gegenüber einer Präsenzveranstaltung. Allerdings sahen mit etwa 70–80 % die meisten den Lerneffekt als gleich gut oder sogar besser an. Hier zeigten sich vergleichbare Zahlen bei den Aufsplittungen nach Geschlecht, Alter sowie Tätigkeitsfeldern.

Der durch den digitalen Kongress erreichte Lernzuwachs wurde im Mittel mit 2,2 ± 0,9 eingeschätzt (5er-Likert-Skala, sehr hoch [1]–sehr niedrig [5]). Relevante gruppenspezifische Unterschiede zeigten sich nicht. Teilnehmer, die älter waren als 60 berichteten den höchsten (60 bis 69 Jahre bzw. > 70 Jahre 2,0 ± 0,9 und 2,0 ± 1,0) und Naturwissenschaftler den niedrigsten Lernzuwachs (2,7 ± 1,0).

Das „Live“-Session-Format wurde in allen Gruppen (Geschlecht, Alters- bzw. Berufsgruppe, Tätigkeitsfeld) als das Format angesehen, welches den größten persönlichen Lernzuwachs ermöglicht. Erwähnenswert ist, dass das On-demand-Format v. a. von Studierenden und Naturwissenschaftlern bevorzugt wurde (11,8 und 19,2 %). Das „Fishbowl“-Format wurde von 16,7 % der Vertreter der pharmazeutischen Industrie und 7,7 % der Naturwissenschaftler favorisiert.

Die zeitliche und örtliche Flexibilität des virtuellen Kongresses gegenüber dem herkömmlichen Format wurde von insgesamt 94,1 % als „besser“ oder „gleich gut“ bewertet, unabhängig vom Tätigkeitsfeld und der Berufsgruppe. Vor allem Praxen und die Gruppe der 30- bis 39-Jährigen bewerteten die zeitliche und örtliche Flexibilität als „besser“.

Eine deutliche Mehrheit der Teilnehmer (85,3 %) würde es begrüßen, die Kongressinhalte dauerhaft „on demand“ abrufen zu können. Dabei fielen keine signifikanten Unterschiede bezüglich Geschlecht, Alter oder Tätigkeitsfeld auf. Dieser Wunsch war bei RFA/MFAs mit 69,4 % am wenigsten ausgeprägt.

Die Mehrheit (80,4 %) war mit der Veranstaltung insgesamt „zufrieden“ oder „sehr zufrieden“. Im Mittel wurde der virtuelle Kongress auf einer 5er-Likert-Skala (sehr zufrieden [1] – sehr unzufrieden [5]) mit 2,0 ± 0,9 bewertet. Die Bewertung zeigte sich unabhängig von Altersgruppe und Geschlecht. Naturwissenschaftler und Labormitarbeiter bewerteten sie mit im Mittel 2,3 ± 1,1 bzw. 2,5 ± 1,7 am schlechtesten. Zwischen (Uni‑)Klinikern und in Praxen Tätigen zeigten sich keine signifikanten Unterschiede bezüglich der Zufriedenheit (2,0 ± 0,9 und 1,9 ± 0,9). Auch die Vertreter der pharmazeutischen Industrie waren mit 2,2 ± 1,0 mit dem Kongress zufrieden. Studierende bewerteten den virtuellen Kongress mit 1,5 ± 0,5 am besten.

Die ausgewählten Themen des Kongresses entsprachen den Erwartungen von 89,1 % der Teilnehmer (Angaben „trifft zu“/„trifft eher zu“). RFA/MFAs (80,0 %) und andere (78,9 %) stimmten dieser Aussage in geringerem Ausmaß zu. Ein relevanter Unterschied zeigte sich für Frauen (88,7 %) und Männer (90,4 %) nicht.

Die präsentierten Inhalte wurden von 85,6 % der Teilnehmer als für ihre Tätigkeit relevant eingestuft. In der Altersgruppe < 40 Jahre lag dieser Wert mit 79,2 % niedriger. Geschlechtsspezifische Unterschiede oder Unterschiede zwischen Klinikern (86,0 %) und Praxen (86,9 %) zeigten sich nicht.

Für 62,7 % bestand ausreichend Gelegenheit für Fragen und Diskussionen. Relevante Unterschiede zeigten sich auch für dieses Item bezüglich Geschlecht und Tätigkeitsfeld nicht. Die Altersgruppe der über 60-Jährigen stimmte der Aussage am häufigsten zu (70,5 %).

**Figure 2:**
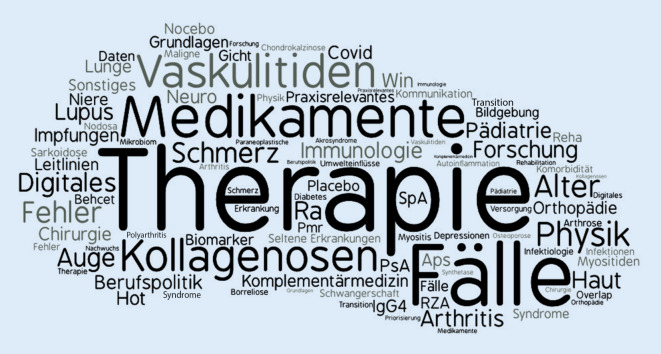

Aus den Freitextangaben wurden die für den Jahreskongress 2021 gewünschten Themengebiete identifiziert und nach Häufigkeit inhaltlich gruppiert. Eine visuelle Darstellung dieser Freitextangaben zeigt die „Wortwolke“ in der Abb. 2.

## Diskussion

Das virtuelle Format „Deutscher Rheumatologiekongress“ wurde zum Erfolg. Fast 2600 Teilnehmer belegen das ungebrochene Interesse an der Jahrestagung. Diese Zahlen lagen in den vergangenen Jahren mit 2500 bis maximal 2800 Besuchern in demselben Bereich. Der individuelle Lernerfolg wurde auch mit dem neuen, noch ungewohnten Format als hoch eingeschätzt.

Die Ergebnisse dieser Arbeit geben Aufschluss darüber, wie die Deutsche Gesellschaft für Rheumatologie virtuelle oder hybride Konferenzen in der Zukunft gestalten kann und sollte, um auf die Interessen der Teilnehmer bestmöglich eingehen zu können. Die im Wesentlichen positiven Rückmeldungen zur Technik lassen erkennen, dass diese kein Hindernis mehr in der Umsetzung von Online- bzw. hybriden Kongressen sein dürfte. Das für das Pandemiejahr 2020 zwangsweise gewählte virtuelle Format des Kongresses wurde von einer deutlichen Mehrheit der Teilnehmerinnen und Teilnehmer sehr gewürdigt. Dieses Ergebnis ist insbesondere aufgrund der sehr kurzen Vorbereitungszeit für die inhaltliche und v. a. komplett neue organisatorische Gestaltung des Kongresses seitens der DGRh und der Rheumaakademie hoch einzuschätzen.

Die inhaltlichen Bewertungen der Evaluation zeigten, dass die ausgewählten Themen und die Umsetzung in den ausgewählten Session-Formaten des Kongresses bei den meisten Teilnehmern auf Interesse stießen. Im Gegensatz zu Klinikern und in Praxen Tätigen wurden Bedürfnisse von Naturwissenschaftlern und Studierenden nur partiell abgedeckt. Letztere bewerteten den Kongress dennoch positiv. Sie profitieren offenbar als Mentees von Vor-Ort Kongressen durch die enge persönliche Begleitung und dadurch auch inhaltliche Führung durch die Mentoren sicherlich mehr, als dies in einem Online-Format umsetzbar ist. Auch die Gruppe der unter 40-Jährigen berichtete einige Defizite, wie die Relevanz der Themen für ihre Tätigkeit. Hier gilt es, entsprechende Inhalte näher zu identifizieren und anzubieten. Aufgrund der multiplen Freitextangaben konnten Themengebiete identifiziert werden, die von den Teilnehmern als relevant für kommende Kongresse angesehen werden. Diese werden bereits in den Planungen für das Jahr 2021, die Anfang Oktober 2020 in einer weiteren Detailtiefe gestartet sind, berücksichtigt. So kann ein Jahreskongress 2021, der sich möglichst nah an den Bedürfnissen seiner Teilnehmer orientiert, noch effektiver gestaltet werden.

Die Möglichkeit, sich weitere Sessions des Kongresses im Rahmen des On-demand-Angebotes ansehen zu können, wurde von den Teilnehmern sehr geschätzt, und es bestand Interesse daran, Kongressinhalte auch nach dem Kongress abrufen zu können. Diese Funktionalität war in 2020 aus verschiedenen Gründen noch nicht durchgängig für alle Kongressinhalte gegeben und wurde vonseiten der Kongressorganisation auf einen Zeitraum bis zum 31.12.2020 beschränkt. Hier müssen weitere Zugriffsanalysen erfolgen und Fragen zu Autorenrechten und Lizenzen geklärt werden, bevor z. B. bestimmte Kongressbeiträge langfristig in Form einer Mediathek verfügbar gemacht werden können. In diesem Zusammenhang sind auch Themen zu sehen, die durch das virtuelle Format erst entstehen, wie z. B. Aufklärung der Präsentierenden über (Lizenz-)rechtliche Probleme und Online-Hilfestellungen für Präsentierende.

Der Wegfall der räumlichen Trennung von (virtueller) Fortbildung und realem privatem oder beruflichem Aufgabenfeld bietet zwar den Vorteil einer höheren Flexibilität, u. A. wegen des entfallenden Aufwandes für An- und Abreise. Andererseits dürfte die örtliche Verfügbarkeit für wichtige private oder berufliche Aufgaben zu einer Konkurrenz mit virtuellen Fortbildungen führen, die auch durch asynchrone On-demand-Angebote nicht wirklich kompensiert werden kann [2, 5]. Demgegenüber erfordern Live-Chats und Fishbowl-Diskussionsrunden auch bei einem virtuellen Kongress eine synchrone (zeitgleiche) Kommunikation wie bei einer Präsenzveranstaltung. Hier bestand nach dieser Evaluation des virtuellen DGRh-Kongresses für etwa 2/3 der Teilnehmer ausreichend Gelegenheit für Fragen und Diskussionen.

Diese zunächst rein theoretischen Überlegungen zur Kommunikation und zu virtuellen Fortbildungsformaten könnten zukünftig Einfluss auf die Gestaltung und Organisation von Kongressen, v. a. bei einer sog. „hybriden“ Form sein, bei der man genau entscheiden muss, welches Format eines Fortbildungsangebotes für welche Informationsübermittlung auch oder am besten geeignet ist.

Die Interaktivität und Anpassung der Vorträge an das neue Format blieb bis auf wenige Vorschläge – wie es z. B. das Format Fishbowl aufgrund seiner Struktur vorgibt – den Präsentierenden überlassen. Daraus resultierten überwiegend klassische, z. T. sogar vollständig voraufgezeichnete Vorträge (d. h. in der Regel Screencasts). Solche On-demand-Inhalte bieten von vornherein keine Interaktivität. „Echte“, sprachliche Interaktivität mit dem Publikum war im Kongress daher nur bedingt gegeben, sie kann durch die moderierten Chat-Funktionen auch nur teilweise kompensiert werden. Ein „ständiges Rederecht für alle Teilnehmenden“ stellt keine Option dar, da dann ggf. ohne Rücksicht auf andere gleichzeitig gesprochen wird. Konkrete Forderungen und Hilfestellungen bei der Nutzung interaktiver Möglichkeiten, moderner Präsentationen, in Echtzeit ausgewertete Publikums-Polls o. Ä. sind sinnvolle Optimierungen digitaler Session-Formate. Diese sind bei der inhaltlichen und v. a. technischen Planung der Veranstaltung zu berücksichtigen und sollten Präsentierenden als moderne Optionen offeriert werden. Hier ist die Aussage der M‑Events Cross Media GmbH von Bedeutung, wonach bislang nur wenige Kongresse dieser Größenordnung ein vergleichbares, geschweige denn höheres Maß an Interaktivität boten.

Wie erwartet, wurde als Limitation der fehlende kollegiale Austausch benannt, auch wenn Public-Viewing-Aktivitäten in 6 DGRh-Rheumazentren dieser hatten entgegenwirken wollen. Die Resonanz der Teilnehmer vor Ort war in persönlichen Gesprächen durchweg positiv, wobei eine strukturierte Evaluation aufgrund der kurzfristigen Umsetzung der Veranstaltungen nicht erfolgen konnte.

Auf spezifische Evaluationen einzelner Sessions wurde verzichtet, um die freiwillig Teilnehmenden nicht mit weiteren Fragen zu überfrachten. Eine solche ist aber sicher sinnvoll, um Vor- und Nachteile einzelner Session-Formate, Interesse an bestimmten Themen und organisatorische Optimierungsmöglichkeiten zu identifizieren. Hierfür bieten sich zukünftig digitale On-the-fly-Befragungen der Teilnehmer während bzw. direkt nach der Veranstaltung an.

Eine „Digitalisierung“ wissenschaftlicher rheumatologischer Kongresse hatte bis zur COVID-19-Pandemie „nur“ durch die zunehmende Nutzung verschiedener Social-Media-Kanäle (z. B. Facebook, Twitter) stattgefunden. Es wird ihnen dabei eine relevante Aufgabe in der Wertschöpfung zugeschrieben, indem kurze Videoproduktionen, die über wichtige Studien und Ereignisse des Kongresses berichten und überwiegend von der pharmazeutischen Industrie vorangetrieben wurden, veröffentlicht wurden [6]. Im Rahmen des virtuellen Deutschen Rheumatologiekongresses waren die Einträge auf dem DGRh-Facebook-Account noch spärlich, der Twitter-Account der AG junge Rheumatologen wurde häufiger genutzt. Hier konnte somit Nachholbedarf identifiziert werden.

### Schlussfolgerung

Zusammenfassend kann festgestellt werden, dass die COVID-19-Pandemie und die bestehenden Reise- und Kontaktbeschränkungen dazu beigetragen haben, auch für die deutsche Rheumatologie neue Wege der Interaktion und Kommunikation für Kongresse, Fortbildungsveranstaltungen und weitere Formen des Wissensaustausches zu identifizieren und erfolgreich umzusetzen. Abzuwarten bleibt, ob die virtuellen Formate nur als vorübergehende Entwicklungen zu betrachten sind oder ob sie sich zu einem dauerhaften, zunehmend noch besser organisierten Instrument des Wissensaustausches entwickeln, ggf. auch nur als „Add-on“ im Sinne hybrider Veranstaltungsformate [7].

Direkte persönliche Begegnungen scheinen trotz aller Vor- und Nachteile virtueller Fortbildungsformate dennoch unverzichtbar, da der persönliche Austausch mit Kollegen von allen Teilnehmergruppen vermisst wurde, auch wenn ökonomische und ökologische Aspekte ein rein virtuelles Format priorisieren mögen.
